# Vertebral cryptococcosis: An uncommon cause of a paravertebral mass

**DOI:** 10.1590/0037-8682-0353-2019

**Published:** 2020-01-27

**Authors:** Igor Biscotto, Miriam Menna Barreto, Rosana Souza Rodrigues, Edson Marchiori

**Affiliations:** 1 Universidade Federal do Rio de Janeiro, Departamento de Radiologia. Rio de Janeiro, RJ, Brasil.; 2 Instituto D’Or de Pesquisa e Ensino, Rio de Janeiro, RJ, Brasil.

**FIGURE 1:**
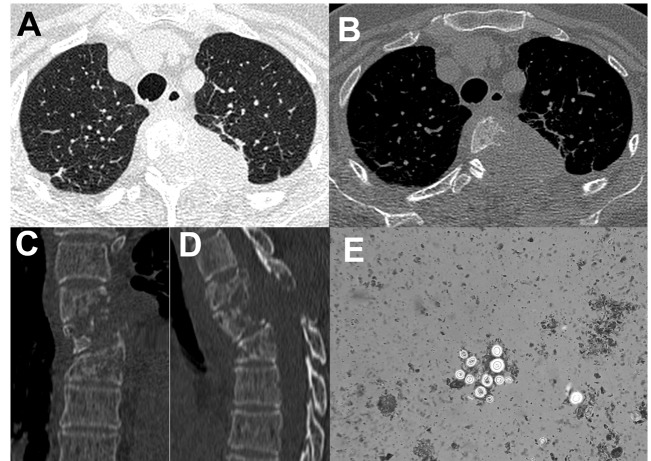
Chest CT with axial images of the lungs **(A)** and the bone window **(B)** show a soft-tissue mass in the left paravertebral region, with extension to the adjacent vertebra and the posterior costal arch. Coronal **(C)** and sagittal **(D)** reformatted images show lytic lesions involving the T3-T5 vertebral bodies. **(E)** CT-guided biopsy reveal multiple round, thin-walled, encapsulated yeast cells (*C. neoformans*), identified by India ink staining.

A 55-year-old female patient presented to our hospital with a 4-month history of progressive paraparesis. She experienced progressive dyspnea and a 30kg-weight loss during this period but no fever, night sweats, or cough. Physical examination revealed bilateral lower extremity weakness and multiple skin lesions on the face and neck, characterized by umbilicated papules. Chest computed tomography (CT) revealed a paravertebral soft tissue mass with hypodense foci, extending between the T3-T5 vertebrae on the left side. The mass occupied the T3-T4 and T4-T5 neural foramina bilaterally, with spinal cord compression at these levels (Figure 1A and Figure B). Multiple lytic lesions were noted on the T3-T5 vertebral bodies and pedicles, and posterior costal arches, predominantly on the left side (Figure 1C and Figure D). Her hemogram revealed lymphopenia (600 lymphocytes/mm^3^). Serological tests were positive for HIV infection; the CD4 count was 40 cells/mm^3^. A CT-guided needle biopsy and India ink staining detected *Cryptococcus neoformans* characterized by multiple, round, thin-walled, encapsulated yeast cells (Figure 1E). Culture and latex agglutination test for fungi confirmed the presence of *Cryptococcus*. Biopsy of the skin lesions and cerebrospinal fluid cultures were positive for *C. neoformans.* The patient died 2 months following diagnostic confirmation despite treatment with amphotericin B and fluconazole.

Vertebral involvement in cryptococcal infection is extremely rare. The clinical symptoms and radiological findings of skeletal cryptococcosis are nonspecific. Histopathology and detection of fungi in the lesions confirm the diagnosis^1^ ^,^ ^2^ ^,^ ^3^.
